# Supplementary material for: A Review of Published Literature Regarding Health Issues of Coastal Communities in Sabah, Malaysia
Source: Int J Environ Res Public Health. 2020 Feb 27;17(5):1533. doi: 10.3390/ijerph17051533 (PMC7084212; doi:10.3390/ijerph17051533)
Supplement: Supplementary file 1 [file ijerph-17-01533-s001.pdf]

International Journal of
Environmental Research and Public Health

MDPI

Table 1. Summary table of studies included in the present systematic review.

**Title of the article (year of publication)**	**Study objective(s)**	**Study methodology**	**Health status measured**	**Health status reported**	**Quality assessment**
Epidemiology of *Plasmodium knowlesi* malaria in north-east Sabah, Malaysia: Family clusters and wide age distribution (2012)	To determine the epidemiological burden of *Plasmodium knowlesi* at Kudat, Sabah<br>To determine the association between malaria cases and rainfall events at Kudat	Retrospective cross-sectional study design with quantitative method<br>Study was conducted at Kudat<br>Data were collected in all positive malaria cases at Kudat Hospital from January 2009 to November 2011 ($n = 653$)	Incidence of malaria infection at Kudat<br>Distribution of cases according to age and family cluster	The incidence of malaria was found to be 2.6/1000 people/year, and *Plasmodium knowlesi* was observed in 1.4/1000 people/year<br>The most common malaria species at Kudat was *Plasmodium knowlesi*<br>This study found that malaria affects all ages from young children to the elderly and men and women similarly	77%
Individual-level factors associated with the risk of acquiring human *Plasmodium knowlesi* malaria in Malaysia: A case-control study (2017)	To evaluate the potential exposure risk associated with *Plasmodium knowlesi* malaria	Case-control study design with quantitative method<br>Study was conducted at Kudat and Kota Marudu<br>Data were collected from December 2012 to December 2014<br>'Case': a patient with positive malaria infection who was febrile and lived in the study area for the past 3 weeks during the data collection period ($n = 320$)<br>'Control': individual who lived in the study area for the past 3 weeks and did not have fever during the data collection period ($n = 953$)	Risk factors such as demographic, behavioural, and socioeconomic factors, housing condition, red blood cell polymorphism, serological and immunological blood markers were compared between the case and control patients.	Age 15 years or older, male sex, working a at plantation site, history of outdoor activities, being aware of the presence of monkeys in the area, and having open eaves or gaps in walls were independently associated with increased risk of symptomatic *Plasmodium knowlesi* infection<br>Known case of glucose-6-phosphate dehydrogenase deficiency, farming as an occupation, and having long grasses around the house were significantly associated with *Plasmodium knowlesi* infection but not with other *Plasmodium* species infections	95%

Title of the article (year of publication)	Study objective(s)	Study methodology	Health status measured	Health status reported	Quality assessment
Changing epidemiology of malaria in Sabah, Malaysia: Increasing incidence of *Plasmodium knowlesi* (2014)	To describe the changes in epidemiology of malaria in Sabah from one species to another species of *Plasmodium*<br>To determine whether the increasing incidence of *Plasmodium knowlesi* represents microscopic misdiagnosis or increased recognition of *Plasmodium knowlesi*<br>To describe the age and sex distribution of malaria infection<br>To determine the association between malaria notification and rainfall events	Retrospective cross-sectional study design with quantitative method<br>Study reported data for Kudat, Keningau, Ranau, Tawau, Sandakan, and Kota Kinabalu<br>Malaria notification data were collected from 2007 to 2013<br>Demographic and epidemiological information of infected individuals were also collected from the surveillance<br>Rainfall data were obtained from January 2009 to 2012 for Kudat, Keningau, Ranau, Tawau, Sandakan, and Kota Kinabalu	Malaria infection notification rate and species of *Plasmodium* reported<br>Association between age and sex with malaria infection	Notifications rate of *Plasmodium malariae*/*Plasmodium knowlesi* increased from 703 in 2011 to 815 in 2012 and 996 in 2013<br>Majority of malaria notifications was caused by *Plasmodium malariae*/*Plasmodium knowlesi* accounting for 62% of all malaria notifications in 2013<br>Plasmodium polymerase chain reaction testing among samples diagnosed with *Plasmodium malariae*/*Plasmodium knowlesi* infection showed that 85% of the cases were *Plasmodium knowlesi* monoinfection. This demonstrates that the increase in notifications is likely to represent a true increase in the incidence of *Plasmodium knowlesi* rather than the microscopic misdiagnosis of *Plasmodium* species	82%
Association between landscape factors and spatial patterns of *Plasmodium knowlesi* infections in Sabah, Malaysia (2016)	To assess the potential associations between *Plasmodium knowlesi* incidence and environmental variables derived from satellite-based remote-sensing data	Retrospective cross-sectional study design with quantitative method<br>Study was conducted at Kudat and Kota Marudu<br>Data were collected for all positive malaria cases from 2008 to 2012 ($n = 739$)<br>Data for environmental variables were collected from various datasets derived from satellite-based remote-sensing data	Incidence of *Plasmodium knowlesi* infection<br>Association between the environmental factors and *Plasmodium knowlesi* incidence	The range of the estimated annual parasitic incidence expressed as cases/1000 person/y) for *Plasmodium knowlesi* malaria was approximately 102	86%
Plasmodium knowlesi malaria in children (2011)	To describe the demographic, clinical, and laboratory features of *Plasmodium knowlesi* infection in children in Kudat, Sabah	Retrospective case-series study design with quantitative method<br>Study was conducted at Kudat district hospital<br>Data were collected for all children (paediatric cases) admitted with positive malaria cases from January to November 2009 ($n = 41$)	Demographic characteristic of children with *Plasmodium knowlesi* malaria<br>Clinical features of *Plasmodium knowlesi* and *Plasmodium falciparum* malaria<br>Laboratory features of *Plasmodium knowlesi*—anaemia, thrombocytopaenia<br>Response to treatment	The incidence of *Plasmodium knowlesi* malaria was higher in men than in women<br>Overall mean age of the children: 8.9 years<br>Children infected with *Plasmodium falciparum* malaria were significantly younger than children infected with *Plasmodium knowlesi* infection<br>Clinically, the duration of fever was shorter with *Plasmodium knowlesi* malaria than with *Plasmodium falciparum*; however, it was not statistically significant	86%

**Title of the article (year of publication)**	**Study objective(s)**	**Study methodology**	**Health status measured**	**Health status reported**	**Quality assessment**
Pulmonary tuberculosis in outpatients in Sabah, Malaysia: advanced disease but low incidence of HIV co-infection (2015)	To describe the epidemiological characteristics of patients with tuberculosis (TB)<br>To determine the prevalence of human immunodeficiency virus (HIV) co-infection<br>To assess the sensitivity and specificity of the locally available point-of-care HIV test kits	Cross-sectional study design with quantitative method<br>Study was conducted at Luyang TB clinic in Kota Kinabalu<br>Data were collected for all smear-positive pulmonary TB patients who were 15 years old and above from July 2012 to July 2014 (n=176)	Demographic characteristics of TB patients<br>Patients' clinical characteristics and comorbidities	Demographic characteristics: 59% of the patients were men with median age of 30 years. A total of 67% were Malaysians, and 33% were foreigners or stateless.<br>Clinical characteristics and comorbidities among the patients: diabetes mellitus (6.8%), hypertension, (5.1%), HIV infection (1.7%), smokers (22.7%), ex-smoker (28%), and past TB (7.4%).<br>Radiological severity: cavitary disease (65%)<br>Smear grade: scanty (17.0%), 1+ (28.9%), 2+ (25.2%), and 3+ (28.9%)<br>Haemoptysis (46.0%)	91%
Understanding tuberculosis: perspectives and experiences of the people of Sabah, East Malaysia (2010)	To assess the knowledge and perceptions of TB patients and the community about TB<br>To assess the experiences of healthcare services<br>To examine the impact of TB on patients and families	Qualitative study design through in-depth interviews<br>Study was conducted in seven districts (Kota Kinabalu, Penampang, Putatan, Tuaran, Kota Marudu, Kudat, and Keningau)<br>Interviews were conducted on TB patients, other non-patient respondents (who were either spouses, relatives, or other people in the same village as the patients), and healthcare personnel in the districts (n=58)	Knowledge and perceptions of TB disease<br>Health-seeking behaviours of the patients	A total of 96% of the respondents (TB patients) did not know the actual cause of TB. They believed it was due to stress, contaminated food, exposure to rain, sharing utensils with other infected patients, and hereditary or genetic<br>A total of 98% of the patients believed that TB is not an infectious disease<br>A total of 74% of the patients reported that they only sought treatment due to worsening of symptoms such as following haemoptysis or shortness of breath<br>Majority of the patient often felt weak and never fully recovered to their pre-illness physical state<br>Following the diagnosis of TB, patients changed their life practices—not sharing their utensils, had a separate sleeping area, and practised social distancing<br>TB causes significant burden to the patient due to stigmatisation<br>A total of 91% of patients reported that the healthcare services provided for TB were good<br>Majority of healthcare workers were unaware that the knowledge regarding TB was insufficient, and they did not know the effects of TB on their patients. This was mainly due to limited discussion between the health staff and the patients on the impact of TB	95%
An outbreak of tetrodotoxin poisoning from consuming horseshoe crabs in Sabah (2017)	To describe the case series of tetrodotoxin poisoning in order to determine the risk factors to prevent further outbreaks	Case report study with quantitative method<br>Study was conducted at Kota Marudu Hospital<br>Data were collected for all positive and possible cases from June to August 2011 (n = 30)	Number of cases reported and the socio-demographic and clinical characteristics of the patient	Patients with tetrodotoxin poisoning presented with several manifestations such as dizziness (80%), circumoral and lingual numbness (80%), hand and feet numbness (63.3%), nausea and vomiting (30%), and weakness and difficulty in breathing (26.6%)<br>Three patients (10%) died, while 27 patients recovered. Forty-seven per cent of the patients had onset of symptoms within 30 minutes of ingestion of horseshoe crabs	100%

Title of the article (year of publication)	Study objective(s)	Study methodology	Health status measured	Health status reported	Quality assessment
Case report: Paralytic shellfish poisoning in Sabah (2017)	To understand paralytic shellfish poisoning (PSP) outbreak cases in Sabah and making future informed decisions to prevent it	Case report study with quantitative method<br>Study area included all cases from coastal areas such as squatter area in Taman Jaya Diri, Sepanggar, Kota Kinabalu and Tuaran, Numbak village, Sepangar and Rancangan Mawao village, Membakut<br>Data were collected from respective hospitals that reported positive cases of PSP. The hospitals included were Queen Elizabeth Hospital in Kota Kinabalu, Tuaran Hospital, and Beaufort Hospital<br>Data were collected for 6 months from January to June 2013 ($n = 58$)	Socio-demographic and clinical characteristics of the patients were recorded<br>Information on their ethnicity, age, sex, occupation, residence, shellfish consumed,<br>symptoms, and onset of the symptoms were recorded	Of the 58 patients (24 men and 34 women), only 24 were Malaysians. The others were from neighbouring countries<br>The overall mean age of all cases was 30.7 years<br>Among the victims, five were less than 10 years old (the youngest was 8 years old), while the six patients were greater than 50 years old (the oldest was 67 years old). The highest percentage was recorded for 11–20-year age group<br>Of the positive 58 patients, 44 were admitted to the hospitals, and four of the patient died due to PSP<br>The remaining 14 patients received outpatient treatment due to dizziness and vomiting<br>The most common symptoms shown by the patients were circumoral, lingual, and neck numbness (83%), whereas breathing difficulty and faintness were presented by few patients.<br>Approximately 35% of the patients also experienced nausea, and 9% of the patients fainted	100%
Iodine status among pregnant women in rural Sabah, Malaysia (2017)	To evaluate the iodine status of pregnant women living in several selected rural divisions in Sabah 13 years after the implementation of the mandatory universal salt iodisation programme	Cross-sectional study design with quantitative method<br>Study was conducted at three rural divisions of Sabah (the Interior, the West Coast, and Kudat)<br>Data were obtained through<br>(i) face-to-face interviews, (ii) physical examination of the neck, and (iii) urine analysis<br>Data were collected from all pregnant women attending the government maternal and child health clinics from early May to end of June 2013 ($n = 524$)	Data on the date of birth, ethnicity, nationality, gestational week, gravida, weight, and height were obtained<br>The respondents were asked about their domestic iodised salt use and iodine- containing supplement consumption<br>Neck examination was performed by trained nurses, and if neck enlargement was observed, it was graded based on the international classification<br>Urine analysis was performed to examine urinary iodine concentration (UIC)	Of all the included patients, five of them (1%) had an enlarged thyroid, indicating iodine deficiency disorder; four had grade 1 goitre, and one had grade 2 goitre<br>Regarding geographical location, three patients with grade 1 goitre were living in the West Coast, and the remaining two were living in Kudat<br>The median UIC among the participants was 105 μg/L<br>More than half (60.5%) of the women had a UIC <150 μg/L (insufficient iodine), 22.8% had a UIC of 150–249 μg/L (adequate iodine), and 16.6% had a UIC ≥ 250 μg/L (more than adequate or excessive iodine)<br>The median UICs differed significantly between all three divisions, and Kudat had significantly higher UICs than those from the Interior	90%

**Title of the article (year of publication)**	**Study objective(s)**	**Study methodology**	**Health status measured**	**Health status reported**	**Quality assessment**
Antenatal care practice and pregnancy outcome at Kudat area, Sabah, Northern Borneo (2017)	To assess the antenatal care (ANC) related knowledge<br>To assess the antenatal practice<br>To assess the outcome of pregnancy	Cross-sectional study design with quantitative method<br>Study was conducted among community villages in Kudat<br>Data were obtained through face-to-face interviews using a semi-structured questionnaire<br>Data were collected from women who were ever-married and having at least one pregnancy experience in 5 years and resided in the villages at Kudat area<br>Data were collected from December 2014 to October 2016 ($n = 300$)	Level of knowledge and practice on ANC care and outcomes of pregnancies	Among the study participants, 53% of them had insufficient knowledge, in contrast to 47% with sufficient knowledge on ANC<br>Nearly all of the respondents received antenatal care (99%), and they were encouraged to receive ANC by the healthcare providers<br>ANC was started to be obtained at 1 month (11%), at 2 months (36%), and at 3 months (40%), and late ANC was reported by 13% of the respondents<br>The mean ANC visit was 9 times, and greater than 80% of the respondents received all types of ANC services<br>All deliveries were performed in the hospitals (97%) and health clinics (3%). Approximately 92% of pregnant women did not experience maternal and infant complications	86%
